# Attributes of context relevant to healthcare professionals’ use of research evidence in clinical practice: a multi-study analysis

**DOI:** 10.1186/s13012-019-0900-8

**Published:** 2019-05-22

**Authors:** Janet E. Squires, Laura D. Aloisio, Jeremy M. Grimshaw, Kainat Bashir, Kristin Dorrance, Mary Coughlin, Alison M. Hutchinson, Jill Francis, Susan Michie, Anne Sales, Jamie Brehaut, Janet Curran, Noah Ivers, John Lavis, Thomas Noseworthy, Jocelyn Vine, Michael Hillmer, Ian D. Graham

**Affiliations:** 1 Department of Health Sciences, School of Nursing, University of Ottawa, Ottawa, ON Canada; 2 Clinical Epidemiology Program, Ottawa Hospital Research Institute, Ottawa, ON Canada; 3 Faculty of Medicine, University of Ottawa, Ottawa, ON Canada; 4 Institute of Health Policy, Management, Evaluation, University of Toronto, Toronto, Canada; 5 Monash Health, Melbourne, Australia; 6 Deakin University, Geelong, Australia; 7 City University of London, London, UK; 8 University College London, London, UK; 9 University of Michigan, Ann Arbor, MI USA; 10 Center for Clinical Management Research, VA Ann Arbor Healthcare System, Ann Arbor, MI USA; 11 School of Epidemiology and Public Health, University of Ottawa, Ottawa, ON Canada; 12 Faculty of Health, School of Nursing, Dalhousie University, Halifax, NS Canada; 13 IWK Health Centre, Halifax, NS Canada; 14 Women’s College Hospital, Toronto, ON Canada; 15 McMaster University, Hamilton, ON Canada; 16 University of Calgary, Calgary, AB Canada; 17 Ontario Ministry of Health and Long-term Care, Toronto, ON Canada

**Keywords:** Context, Secondary analysis, Evidence-based practice

## Abstract

**Background:**

To increase the likelihood of successful implementation of evidence-based practices, researchers, knowledge users, and healthcare professionals must consider aspects of context that promote and hinder implementation in their setting. The purpose of the current study was to identify contextual attributes and their features relevant to implementation by healthcare professionals and compare and contrast these attributes and features across different clinical settings and healthcare professional roles.

**Methods:**

We conducted a secondary analysis of 145 semi-structured interviews comprising 11 studies (10 from Canada and one from Australia) investigating healthcare professionals’ perceived barriers and enablers to their use of research evidence in clinical practice. The data was collected using semi-structured interview guides informed by the Theoretical Domains Framework across different healthcare professional roles, settings, and practices. We analyzed these data inductively, using constant comparative analysis, to identify attributes of context and their features reported in the interviews. We compared these data by (1) setting (primary care, hospital-medical/surgical, hospital-emergency room, hospital-critical care) and (2) professional role (physicians and residents, nurses and organ donor coordinators).

**Results:**

We identified 62 unique features of context, which we categorized under 14 broader attributes of context. The 14 attributes were resource access, work structure, patient characteristics, professional role, culture, facility characteristics, system features, healthcare professional characteristics, financial, collaboration, leadership, evaluation, regulatory or legislative standards, and societal influences. We found instances of the majority (*n* = 12, 86%) of attributes of context across multiple (*n* = 6 or more) clinical behaviors. We also found little variation in the 14 attributes of context by setting (primary care and hospitals) and professional role (physicians and residents, and nurses and organ donor coordinators).

**Conclusions:**

There was considerable consistency in the 14 attributes identified irrespective of the clinical behavior, setting, or professional role, supporting broad utility of the attributes of context identified in this study. There was more variation in the finer-grained features of these attributes with the most substantial variation being by setting.

**Electronic supplementary material:**

The online version of this article (10.1186/s13012-019-0900-8) contains supplementary material, which is available to authorized users.

## Introduction

Context is a frequently neglected issue in the field of implementation science. Many implementation researchers do not account for or even seek to hold constant contextual factors, treating them as confounders when, in fact, these factors should be understood and incorporated into implementation research efforts to achieve clinical effectiveness in real-world settings. As such, context is an important aspect of pragmatic, as opposed to explanatory, research, and since implementation science is inherently pragmatic, contextual factors must be integrated if they are to be effective in improving clinical practice [1]. As a first pass, we define context as factors that are separate from the actual intervention itself and the actors receiving the intervention, but which may nonetheless contribute to the success of the intervention [2, 3]. Adapting interventions to local contexts is an essential part of pragmatic research; unfortunately, research in implementation science often fails to explicitly consider how local context factors shape implementation success [4]. This leads to implementation interventions that are successful in one setting to fail in alternate settings, possibly due to not accounting for the influence of the local context [2, 5]. To increase the likelihood of successful implementation, researchers need to assess and explicitly address contextual barriers and/or facilitators that promote and hinder implementation [5].

For many years, researchers have sought to identify, characterize, and explain the mechanisms associated with contextual factors in implementation science. As a result, large but separate bodies of literature on context in implementation science are beginning to emerge. For example, there are syntheses on the contextual determinants of innovation adoption [6–8], the role of context in quality improvement [5], context attributes related to research utilization [9–11], and the role of context in implementation theory and frameworks [4, 12–15]. While each of these syntheses suggests that context is vital to implementation, there is little agreement across them on the important attributes of context to assess and adapt in implementation research. Furthermore, context may vary by clinical setting and healthcare professional group targeted by an implementation effort. Despite these facts, several studies conducted on context in implementation research have been limited to a single clinical setting or one healthcare professional group at a time. This limits our understanding of the range of contextual attributes that are important for successful implementation. This knowledge is vital for the development of assessment tools to measure context in order to (i) tailor implementation intervention design and delivery, (ii) to better interpret the effects of implementation interventions, and (iii) to pragmatically guide researchers and change agents in their implementation efforts. The purpose of this study was to identify contextual attributes and their features relevant to implementation of research by healthcare professionals and compare and contrast these attributes and features across different clinical settings and healthcare professional roles.

## Methods

### Study design and data collection

We compiled a collection of 11 independent studies investigating healthcare professionals’ perceived barriers and enablers to their use of research evidence in clinical practice. The studies were purposively sampled to maximize the variation in the healthcare setting, country, and participant professional role. These data were collected using similar methods across different countries (Australia and Canada), healthcare professionals (multiple specialities of physicians, nurses, organ donation coordinators), settings (primary care and several hospital settings), clinical behaviors (*N* = 11 behaviors, see Table 1), and types of behavior change (implementation and de-implementation), providing richness not available in any single dataset (Table 1). While most studies included physicians and/or nurses, one study also included organ donation coordinators (in addition to physicians and nurses). Organ donor coordinators in Canada are nurses or other allied health professionals and therefore were deemed eligible for our secondary analysis. All data were collected using semi-structured interview guides informed by the Theoretical Domains Framework (TDF) [16, 17]. The TDF was developed in 2005 by a team of psychological theorists working in collaboration with health service researchers and health psychologists [17]. It synthesizes 33 psychological theories that explain behavior change in 14 theoretical domains. The TDF interview guide questions in the 11 studies were broad and open-ended, allowing healthcare professionals to spontaneously identify instances of context that act as barriers to and/or enablers of their use of research evidence in clinical practice. The different clinical behaviors in these 11 studies were specified with a high level of granularity (see Table 1), but all involved the application of evidence from clinical research and/or clinical guidelines. As a result, these data provide a wealth of information on contextual attributes important to implementation science.

**Table 1:** Dataset characteristics

Dataset/clinical behavior	TACT-A					Sample		
	Target	Act	Context	Time	Actor	*N* = 145	Country	Data collection dates
Hand hygiene	Patients	Hand hygiene	Medical and surgical wards	Before initial contact, after contact, before aseptic procedures, and after bodily fluid exposure	Physicians	12	Canada	Sept 2012–Feb 2013
					Residents	8		
Pre-operative assessment	Patients	Completing an assessment without a routine electro-cardiography	Pre-assessment units	During pre-operative assessments	Anesthesiologist surgeons	11 5	Canada	Sept 2009–Oct 2009
Adult computerized tomography head rule	Patients	Using an adult computerized tomography head rule	Adult emergency room	During emergency room visit for a head injury	Emergency room physicians	8	Canada	Mar 2010–Jun 2010
Child computerized tomography head rule	Patients	Using a child computerized tomography head rule	Pediatric emergency room	During emergency room visit for a head injury	Physicians	10	Canada	Jan 2011–Jul 2011
					Nurses	3		
Donation after cardio-circulatory death	Patients	Donation after cardio-circulatory death	Hospitals that perform organ donation	At circulatory death	Intensivists	12	Canada	Oct 2013–Jul 2014
					ICU nurses	8		
					Organ donation coordinators	9		
Fetal monitoring	Patients	Intermittent auscultation for fetal surveillance	Birthing units	During labor	Labor and delivery nurses	12	Canada	Apr 2010–May 2010
Red blood cell transfusion-2	Patients	Watching and waiting vs. infusing red blood cells	General surgery wards	When patient has borderline hemoglobin	Orthopedic surgeons	12	Canada	Sept 2008–Jul 2009
Red blood cell transfusion-1	Patients	Watching and waiting vs. infusing red blood cells	Intensive care units	When patient has borderline hemoglobin	Intensivists	12	Canada	Apr 2008–Oct 2008
Bone mineral density screening	Patients ≥ 50 years	Order a bone mineral density screen	Physician’s office	At next available appointment when find out about fragility fracture	Family physicians	10	Canada	Sept 2012–Nov 2012
Smoking cessation	Patients	Adherence to a guideline for smoking cessation	Primary care	During patient visit	Family physicians	10	Canada	Mar 2009–Oct 2009
Preconception care guidelines	Patients	Adherence to guidelines for preconception care	Primary care (general practitioner office)	During patient visit	Physicians	3	Australia	Oct 2007–Nov 2007

### Data analysis

Data analysis was managed in NVivo 10 software package [18]. Data were analyzed inductively by several team members [JES, LA, KB, KD, IG] using constant comparative analysis [19, 20]. We analyzed all datasets until data saturation was reached, defined as the point when no new codes emerged after five additional interviews. We consensus coded all interviews in small (less than 10 interviews) datasets. For larger datasets (with more than 10 interviews), a minimum of 50% of the interviews were consensus coded. Data analysis occurred in three phases. In phase 1, we coded the interviews for all utterances of context, defined broadly as any aspect of the physical, social, structural, political, organizational, institutional, or legislative influences on organizations, groups, and individuals. This definition of context guided our approach in that all utterances that could be broadly interpreted as a part of this definition of context were coded. First, three independent coders (JES, KB, KD) examined a single interview in each data set to generate a broad coding scheme. Following generation of the coding scheme, two coders (KB, KD) coded each interview independently and then met to conduct consensus; where consensus could not be reached, a third senior team member (JES, IG) made the final decision. We coded all interviews in a single dataset before coding a different dataset. In phase 2, we organized the codes into “features” of context, later aggregated into larger categories called “attributes” of context. This distinction was analytical rather than empirical or theoretical driven. We assigned each feature and attribute a label and definition for identification purposes. In phase 3, we further analyzed the features and attributes of context by setting (primary care, hospital-medical/surgical, hospital-emergency room, hospital-critical care) and professional role (physicians and residents, nurses, and organ donor coordinators—the majority of whom were nurses). We were not able to examine specialities within roles (e.g., general surgeons versus intensive care doctors) as the sample sizes were small.

## Results

### The sample and data

Characteristics of the 11 datasets comprising the sample for the analysis reported in this paper are summarized in Table 1 using the TACT-A principle as follows: Target (population the behavior is performed towards), Action (act that you plan to intervene upon), Context (the clinical setting), Time (timeframe when the action occurs), and Actor (healthcare professionals performing the behaviors) [20]. The analytic sample reported in this paper comprised 145 (68%) of the 212 available transcripts, which reflects the point when data saturation was reached. The analytic sample represents four healthcare professional groups (physicians (*n* = 105, 72%), nurses (*n* = 23, 16%), organ donor coordinators (*n* = 9, 6%), and residents (*n* = 8, 6%)) and four settings (hospital: emergency room (*n* = 21, 14%), hospital: surgical/medical (*n* = 48, 33%), hospital: critical care (*n* = 53, 37%), and primary care (*n* = 23, 16%)) (Table 1).

### Context attributes and their features

In total, 62 unique *features of context* perceived to act as barriers to and/or enablers of healthcare professionals’ use of research evidence in clinical practice emerged from the 145 interviews. Each feature of context surfaced in at least two interviews and at least two datasets. We grouped the 62 unique features into 14 broader *attributes of context*. The number of features in each attribute varied from 1 (e.g., Collaboration) to 13 (e.g., Resource Access). Table 2 lists the 14 context attributes, a consensus definition of each attribute derived by the research team, its frequency from the secondary analysis, and the number of features within the attribute. Further detail on each attribute is in Additional file 1, including the identity of the features within each attribute, definitions for all features and attributes, an illustrative quote for each feature, and the frequency of each feature overall and within each of the 11 included studies specifically.

**Table 2:** Context attributes and definitions, listed in order of frequency of attribute occurrence (*N* = 145 interviews)

Attribute	*n* (%)	Attribute definition	No. of features	Example feature *Definition*
Resource Access	145 (100)	Access to any resources. This does not necessarily imply the proximity or closeness of such resources, but only their accessibility in the broadest sense (see physical space).	13	Time as a resource *Time considered in economic terms: as it is required for the completion of work tasks and as it is managed by staffing and the arrangement of work.*
Work Structure	142 (98)	The arrangement of tasks, responsibilities, and resources within and between the various teams working in a clinical setting. This includes factors such as the delegation of tasks among supervisors and subordinates; the arrangement of schedules, shifts, and on-call duties; the order of work tasks and procedures; and the management of workloads.	12	Scheduling and shift work *Designated work times, the arrangement of work times among a clinical team (including shift work), and other on-call arrangements.*
Patient Characteristics	136 (94)	The attributes of individuals under medical care or treatment. This code refers to the characteristics of patients when considered as a group rather than as individuals; thus, all sub-codes considered for inclusion here had to be generalizable to a patient population (an attribute that could be potentially measured and aggregated).	2	Demographics *Quantifiable characteristics of a patient population including age, sex, weight, number of illnesses or comorbidities, patient acuity, illness severity, and medication history.*
Professional Role	135 (93)	A set of expectations, both formal and informal, associated with a given clinical occupation.	7	Clinical skill set *The technical competencies, knowledge, and abilities that typify a specific clinical role. These are directly related to, and constituted by, the professional role training received during medical training. However, the Skill Set code is differentiated from Professional Role Training code insofar as Skill Set reflects the active employment of a clinician’s particular skills, rather than their acquisition.*
Culture	117 (81)	The inherited ideas, beliefs, values, and attitudes of a group.	2	Organizational culture *The normative beliefs and shared expectations that govern the work behavior of a clinical team or employees of a healthcare facility. These values and expected behaviors are the product of interactions among system members, along with the influence of their work environment, resulting in a common social structure that exists independently and outside individual team members or workers.*
Facility Characteristics	104 (72)	The attributes of a building or group of buildings designated as a site for providing healthcare. These characteristics include the type of facility (i.e., a hospital, a walk-in clinic, a trauma center.), the volume of patients cared for at that location, the geographic location, the geographic catchment, and the presence or absence of medical trainees.	6	Type of facility *The practice setting where a team of clinicians operates. This can include private clinics, hospitals, nursing care homes, public health practices, and specialty practices.*
System Features	74 (51)	Distinct characteristics of a group of related parts that move or work together in order for a health care region, organization, hospital, or clinical practice to run effectively.	3	Record-keeping *The system by which an organization or institution organizes and maintains patient records or charts.*
Healthcare Professional Characteristics	72 (50)	The attributes of individuals working as providers of medical care. This code refers to the characteristics of individuals when considered as a group rather than as individuals; thus, all sub-codes considered for inclusion here had to be generalizable to a healthcare professional population (an attribute that could be potentially measured and aggregated).	2	Experience *Having knowledge or skill in a particular field, especially a profession or job, gained over a period of time. Often used to compare groups with different experience levels (i.e., junior residents* vs *fellows).*
Financial	66 (46)	Monetary receipts (income) and expenditures (costs) relating to clinical behavior or institutional standards.	4	Funding system *A configuration of services that varies from country to country, but in all cases consists of a financing mechanism, a paid workforce, information on which to base decisions and policies, facilities, and logistics to deliver quality medicines and technologies.*
Collaboration	61 (42)	To work jointly with others (including other organizations) or together especially in an intellectual endeavor	1	Collaboration *Informal communications between team members or other medical professionals that influence the clinical behavior of a healthcare provider.*
Leadership	61 (42)	The direction of a clinical team or management of a healthcare organization.	3	Mentorship *A relationship established between a leader or superior and a subordinate or trainee, characterized by a close, and typically enduring, pedagogical exchange, where the subordinate or trainee learns by observing and regularly communicating with the leader or superior.*
Evaluation	44 (30)	The systematic collection of information about the activities, characteristics, and outcomes of programs, services, policies, or processes, in order to make judgments about the program/process, improve effectiveness, and/or inform decisions about future development.	4	Audit *The official inspection of a division, department, or clinician group, typically by an independent body. An audit is differentiated from more conventional evaluations carried out by an organization during the normal course of operations.*
Regulatory or Legislative Standards	31 (21)	Statutes or principles established and enforced by an agency external to the medical profession. Regulatory or legislative standards are here distinguished from guidelines insofar as these standards are binding, often based on law or remuneration structures, and are outside the control of health organizations.	2	Legal *Established statutes outlining the prerogatives and responsibilities of medical professionals, organizations, and the rights of patients.*
Societal Influences	26 (18)	The general level of social knowledge and attitude as it regards to a particular clinical behavior or procedure. For example, widespread attitudes about organ donation, or a public reaction to a hospital audit as it has been portrayed in the media.	1	Societal influences *The general level of social knowledge and attitude as it regards to a particular clinical behavior or procedure. For example, widespread attitudes about organ donation, or a public reaction to a hospital audit as it has been portrayed in the media.*

Of the 14 attributes of context identified, all were evident across multiple clinical behaviors. Most (12 of 14, 86%) of the attributes of context were common across the majority (at least six of the 11) of clinical behaviors studied; only two attributes of context were found in fewer than six of the clinical behaviors: (1) Leadership (in five clinical behaviors) and (2) Regulatory or Legislative Standards (in 4 clinical behaviors) (see Table 3). Four attributes of context were mentioned in the vast majority (> 90%) of the 145 interviews: (1) Resource Access, (2) Work Structure, (3) Patient Characteristics, and (4) Professional Role. Resource Access was the most commonly reported attribute of context, mentioned in all 145 interviews and containing the highest number of specific features (*n* = 13). The most commonly reported features with this attribute were (1) time as a resource (*n* = 114, 78.6%), (2) access to clinical practice guidelines (*n* = 86, 59.3%), and (3) access to documentation (*n* = 81, 55.9%) (see Additional file 1 for definitions and illustrative quotes for each feature). An illustrative quote for the feature of time as a resource follows:

A lot of us are pretty busy though. It’s probably just a matter of not enough time in the day to follow-up with the way there is just no time.—Family physician, behavior: smoking cessation (interview #4).

**Table 3:**
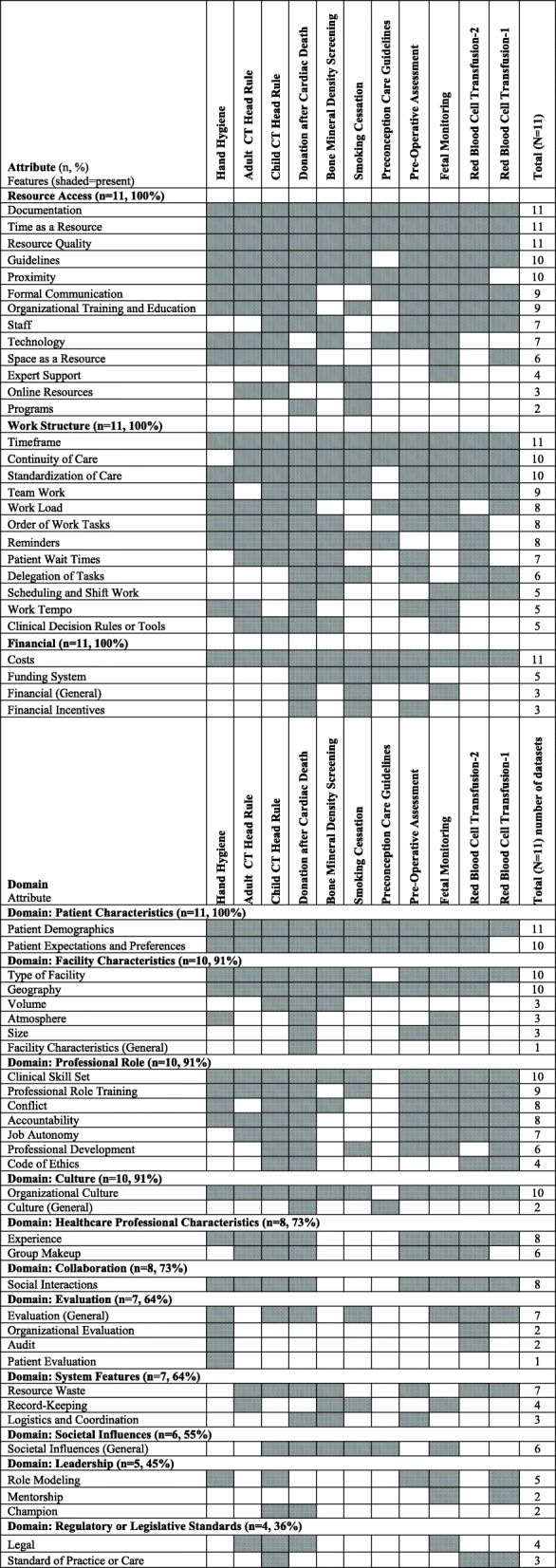
Context attributes and features by clinical behavior

The second most frequently mentioned attribute was Work Structure, mentioned in 142 (97.9%) of interviews and containing 12 specific features. The most common features grouped under Work Structure were (1) timeframe (*n* = 92, 63.4%), (2) continuity of care (*n* = 86, 59.3%), and (3) standardization of care (*n* = 82, 56.6%). An illustrative quote for the feature of standardization of care is:

The issue involves standardization right. So you want to develop a system that can easily followed by many people and applicable across a wide variety of patients.—Anesthesiologist, behavior: routine preoperative assessment (interview #A5).

The third most frequently cited attribute was Patient haracteristics, mentioned in 136 (93.8%) of the interviews and comprising only two specific features: patient demographics (*n* = 113, 77.9%) and patient expectations and preferences (*n* = 90, 62.1%). An illustrative quote for the feature of patient expectations and preferences follows:

… Sometimes patients are adamant that they need the CT head and that would influence [the decision to use the CT Head Rule].—Emergency room physician, behavior: using adult computerized tomography head rules (interview #4).

The fourth most frequently reported attribute, with an overall frequency of > 90%, was Professional Role, mentioned in 135 (93.1%) of interviews and containing seven specific features. The top three features grouped under Professional Role were: (1) clinical skill set (*n* = 75, 51.7%), (2) professional role training (*n* = 55, 37.9%), and (3) interprofessional conflict (*n* = 49, 33.8%). An illustrative quote for this feature is:

It was kind of drilled into me when I was a resident and I see minor head injuries frequently, so it just becomes part of your practice pattern. When you spit out the rules all the time with our residents so it’s just there.—Emergency room physician, behavior: using adult computerized tomography head rules (interview #3)

### Setting

The data used in this secondary analysis came from participants working in primary care and hospitals, which was further divided into four settings for analysis purposes: (1) primary care (*n* = 23, 16%); (2) hospital: medical/surgical units (*n* = 48, 33%); (3) hospital: emergency room (*n* = 21, 14%); and (4) hospital: critical care (*n* = 53, 37%). Table 4 provides a summary of the attributes of context and their features by setting. The shaded areas in Table 4 indicate the presence of the feature. Most context attributes (*n* = 13 of 14, 93%) were mentioned in all four settings with the exception of one attribute, Societal Influences, which was not mentioned by participants in the hospital-surgical/medical setting. When we compared settings at the level of features of context, more variation was evident with only half of the attributes (*n* = 31 of 62, 50.0%) being present in all four settings (Table 4); the remaining 31 attributes varied across settings, however with most features still being relevant to most of the settings. Since we found high consistency in attributes across settings, the summary that follows next identifies some of the *differences* between settings at the feature level. Definitions for each feature along with illustrative quotes are included in Additional file 1.

**Table 4:**
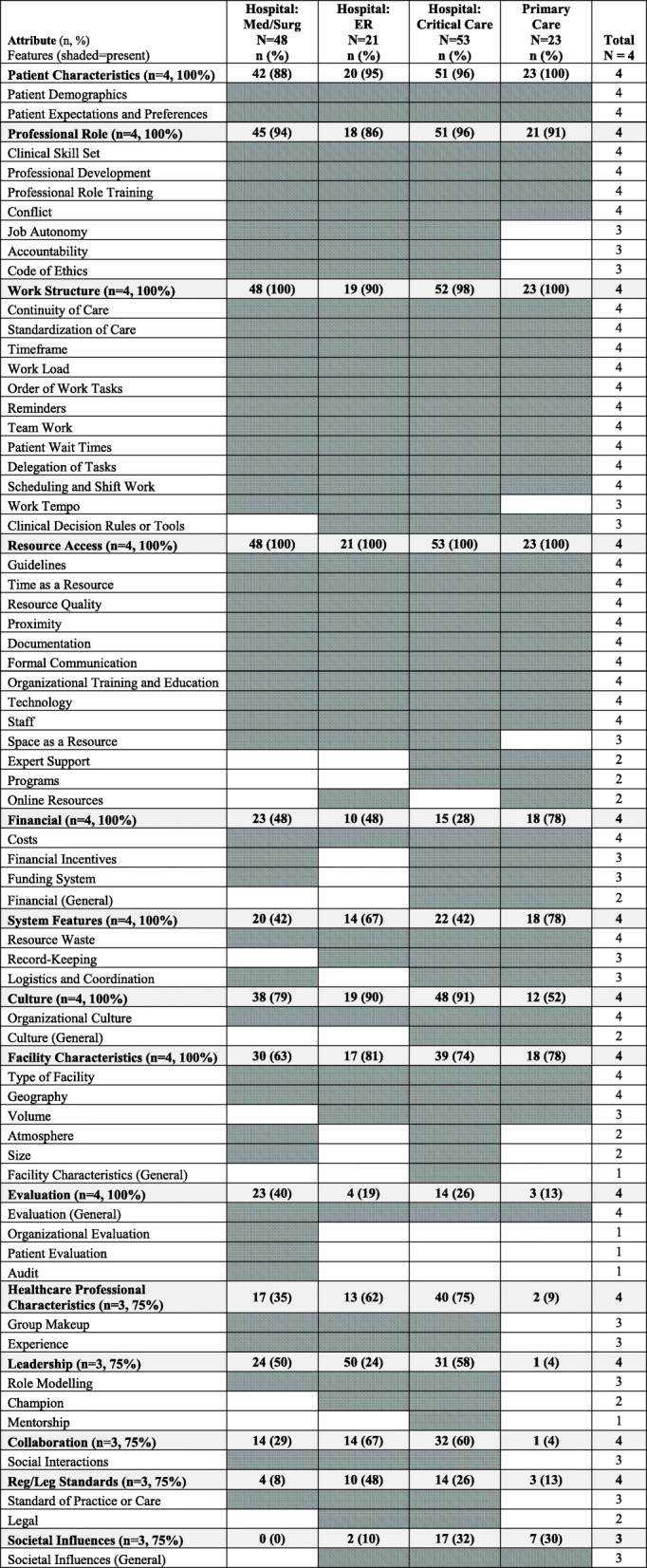
Context attributes and features by setting (*N* = 145 interviews)

#### Primary care

Most context features were relevant to primary care; only six (10%) of the 62 context features were not found in the primary care interviews: (1) atmosphere (attribute: Facility Characteristics), (2) religious affiliation (attribute: Facility Characteristics), (3) experience (attribute: Healthcare Professional Characteristics), (4) mentorship (attribute: Leadership), (5) champion (attribute: Leadership), and (6) organizational evaluation (attribute: Evaluation). Additionally, four other features were more frequent (which we defined as > 25% more mentions in primary care interviews in comparison with interviews from all three hospital-based settings): (1) record-keeping (attribute: System Features), (2) financial incentives (attribute: Financial), (3) programs (attribute: Resource Access), and (4) funding system (attribute: Financial).

#### Hospital: medical-surgical

Most context features were relevant to hospital: medical-surgical settings; only three (5%) of all context features were absent from interviews conducted in hospital-medical/surgical settings: (1) online resources (attribute: Resource Access), (2) team educator (attribute: Resource Access), and (3) societal influences (attribute: Societal Influences). While no features of context were substantially more frequent in hospital medical-surgical settings compared to the other settings, two features were substantially less frequent compared to all other settings: (1) professional development (attribute: Professional Role) and (2) geography (attribute: Facility Characteristics).

#### Hospital: emergency room

Most context features were relevant to hospital: emergency room settings; only 7 (11%) of the 62 context features were absent from interviews conducted in this setting: (1) programs (attribute: Resource Access), (2) team educator (attribute: Resource Access), (3) delegation of tasks (attribute: Work Structure), (4) religious affiliation (attribute: Facility Characteristics), (5) logistics and coordination (attribute: System Features), (6) audit (attribute: Evaluation), and (7) patient evaluation (attribute: Evaluation). One feature was substantially more common in hospital emergency rooms compared to all other settings: resource quality (attribute: Resource Access).

#### Hospital: critical care

Most context features were also relevant to hospital: critical care settings; only one (2%) feature of context was absent from the critical care setting sample: audit (evaluation). Three (5%) additional features however were more frequent in the critical care setting compared to the other settings: (1) conflict (attribute: Professional Role), (2) staff (attribute: Resource Access), and (3) code of ethics (attribute: Professional Role). In addition, two features, were significantly less frequent in the critical care setting: (1) patient wait times (attribute: Work Structure) and (2) reminders (attribute: Work Structure).

### Professional role

We grouped the data into two professional roles: (1) physicians and residents (*n* = 111) and (2) nurses and organ donor coordinators (most of whom were nurses) (*n* = 32). Table 5 provides a summary of the attributes of context and their features by these two roles. Individuals in both professional role groups reported features in all 14 attributes of context. Most (*n* = 11, 79%) attributes of context were reported with similar frequencies (which we defined as less than a 25% difference in frequency by role) across both groups. The three exceptions were (1) Healthcare Professional Characteristics (higher in nurses and organ donor coordinators), (2) Collaboration (also higher in nurses and organ donor coordinators), and (3) Financial (higher with physicians and residents). We saw minimal variation between roles with respect to attributes of context. Only three (5%) features of context surfaced in interviews with one role and not the other: (1) religious affiliation of facility (attribute: Facility Characteristics), (2) funding system (attribute: Financial), and (3) audit (attribute: Evaluation) with each being mentioned by physicians and residents only. Differences in frequency, defined as > 25% difference by role, was identified in 12 (19%) of the features of context. Physicians and residents mentioned two features more frequently: (1) reminders (attribute: Work Structure) and (2) costs (attribute: Financial), while nurses and organ donor coordinators mentioned the remaining 10 attributes more frequently: (1) organizational training and education (attribute: Resource Access), (2) staff (attribute: Resource Access), (3) patient expectations and preferences (attribute: Patient Characteristics), (4) clinical skill set (attribute: Professional Role), (5) conflict (attribute: Professional Role), (6) professional development (attribute: Professional Role), (7) code of ethics (attribute: Professional Role), (8) organizational culture (attribute: Culture), (9) group makeup (attribute: Healthcare Professional Characteristics), and (10) social interactions (attribute: Collaboration).

**Table 5:** Frequency of attributes and features by professional role (*N* = 143 interviews)

Attribute and Feature	Total *N* = 143* *n* (%)	Physicians and residents *N* = 111 *n* (%)	Nurses and organ donor coord. *N* = 32 *n* (%)
Attribute: Resource Access	143 (100)	111 (100)	32 (100)
Time as a resource	112 (78)	88 (79)	24 (75)
Guidelines	84 (59)	59 (53)	25 (78)
Documentation	79 (55)	59 (53)	20 (63)
Proximity	72 (50)	52 (47)	20 (63)
Resource quality	69 (48)	56 (50)	13 (41)
Formal communication	58 (41)	45 (41)	13 (41)
Organizational training and education	50 (35)	28 (25)	22 (69)
Staff	42 (29)	22 (20)	20 (63)
Space as a resource	29 (20)	17 (15)	12 (38)
Technology	28 (20)	18 (16)	10 (31)
Expert support	22 (15)	13 (12)	9 (28)
Programs	12 (8)	9 (8)	3 (9)
Online resources	8 (6)	7 (6)	1 (3)
Team educator	4 (3)	3 (3)	1 (3)
Attribute: Work Structure	140 (98)	108 (97)	32 (100)
Timeframe	90 (63)	67 (60)	23 (72)
Continuity of care	84 (59)	65 (59)	19 (59)
Standardization of care	80 (56)	56 (50)	24 (75)
Team work	68 (48)	48 (43)	20 (63)
Reminders	58 (41)	52 (47)	6 (19)
Work load	36 (25)	22 (20)	14 (44)
Delegation of tasks	36 (25)	25 (23)	11 (34)
Order of work tasks	35 (24)	27 (24)	8 (25)
Work tempo	25 (17)	13 (12)	12 (38)
Scheduling and shift work	21 (15)	12 (11)	9 (28)
Patient wait times	19 (13)	17 (15)	2 (6)
Attribute: Patient Characteristics	134 (94)	103 (93)	31 (97)
Patient demographics	111 (78)	84 (76)	27 (84)
Patient expectations and preferences	90 (63)	61 (55)	29 (91)
Attribute: Professional Role	133 (93)	104 (94)	29 (91)
Clinical skill set	73 (51)	49 (44)	24 (75)
Professional role training	53 (37)	38 (34)	15 (47)
Job autonomy	48 (34)	38 (34)	10 (31)
Conflict	47 (33)	29 (26)	18 (56)
Professional development	43 (30)	27 (24)	16 (50)
Accountability	36 (25)	28 (25)	8 (25)
Code of ethics	30 (21)	15 (14)	15 (47)
Attribute: Culture	115 (80)	86 (77)	29 (91)
Organizational culture	93 (65)	65 (59)	28 (88)
Culture (general)	13 (9)	7 (6)	6 (19)
Attribute: Facility Characteristics	102 (71)	76 (68)	26 (81)
Type of facility	75 (52)	57 (51)	18 (56)
Geography	45 (31)	29 (26)	16 (50)
Size	17 (12)	9 (8)	8 (25)
Volume	14 (10)	12 (11)	2 (6)
Atmosphere	13 (10)	7 (6)	6 (19)
Facility characteristics (general)	10 (7)	7 (6)	3 (9)
Religious affiliation	1 (1)	1 (1)	0 (0)
Attribute: System Features	72 (50)	58 (52)	14 (44)
Resource waste	35 (24)	31 (28)	4 (13)
Logistics and coordination	25 (17)	22 (20)	3 (9)
Record-keeping	24 (17)	14 (13)	10 (31)
Attribute: Healthcare Professional Characteristics	70 (49)	44 (40)	26 (81)
Experience	53 (37)	38 (34)	15 (47)
Group makeup	36 (25)	16 (14)	20 (63)
Attribute: Financial	65 (45)	58 (52)	7 (22)
Costs	58 (41)	52 (47)	6 (19)
Financial incentives	18 (13)	16 (14)	2 (6)
Funding system	12 (8)	12 (11)	0 (0)
Financial (general)	11 (8)	8 (7)	3 (9)
Attribute: Leadership	60 (42)	43 (39)	17 (53)
Role modeling	21 (15)	17 (15)	4 (13)
Mentorship	8 (6)	5 (5)	3 (9)
Champion	6 (4)	5 (5)	1 (3)
Attribute: Collaboration	59 (41)	39 (35)	20 (63)
Social interactions	59 (41)	39 (35)	20 (63)
Attribute: Evaluation	43 (30)	35 (32)	8 (25)
Evaluation (general)	21 (15)	17 (15)	4 (13)
Audit	20 (14)	20 (18)	0 (0)
Organizational evaluation	14 (10)	12 (11)	2 (6)
Patient evaluation	5 (3)	3 (3)	2 (6)
Attribute: Regulatory or Legislative Standards	30 (21)	23 (21)	7 (22)
Legal	20 (14)	13 (12)	7 (22)
Standard of practice or care	13 (9)	11 (10)	2 (6)
Attribute: Societal Influences	25 (17)	13 (12)	12 (38)
Societal influences (general)	25 (17)	13 (12)	12 (38)

*N = 143. Since two of the focus group transcripts contained more than one professional group (physicians and residents), they were analyzed as one transcript, reducing the total number of transcripts when divided by professional role to *N* = 1

## Discussion

### Summary of findings

The purpose of this study was to identify contextual attributes and their features relevant to implementation of research evidence in different clinical settings and healthcare professional roles. We identified 62 unique features of context, which we categorized into 14 broader attributes of context. The 14 attributes of context covered all contextual issues identified in 145 interviews from 11 primary studies of different clinical behaviors. We found instances of all 14 attributes of context across multiple behaviors; most (12 of 14, 86%) of the attributes of context were common across the majority (at least 6) of clinical behaviors. We also found little variation in the 14 attributes of context by setting (primary care and hospitals) and professional role (physicians and residents, and nurses and organ donor coordinators), supporting broad utility of our attributes of context. Most differences in context found were at the finer grained features (of attributes) level, and even then, few features were not relevant to most settings.

### Similarities across behaviors, settings, and professional roles

Five context attributes (Resource Access, Work Structure, Financial, Culture, and Patient Characteristics) were present in all 11 datasets (Table 3) representing all settings and professional roles, suggesting that these attributes may be core contextual factors to be assessed in future implementation research studies. However, these key attributes are largely missing from existing context assessment instruments. Several instruments currently exist and are used widely to examine context generally; for example, the Alberta Context Tool [21], the Context Assessment Index [22], and Organizational Readiness to Change Assessment [23]. Each of these instruments is described in the literature as general measures of context—applicable across various types of behaviors, settings, and roles. The Promoting Action on Research in Health Services (PARiHS) framework was the theoretical basis of each of the instruments. This framework, specifically developed to explain healthcare professionals’ use of research evidence in clinical practice, purports that successful research use is a function of (1) the sources of evidence used to support the practice change, (2) the context (defined as three attributes—leadership, culture, evaluation) where the practice change occurs, and (3) methods used to facilitate the practice change [12, 13]. Our findings suggest that while each of these context attributes (Culture, Leadership, Evaluation) are important, only one of them (Culture) was reported across all behaviors, settings, and professional roles in our analyses. Further, the broad context instruments mentioned above based on the PARiHS framework do not address the majority of other context attributes we identified: for example, Work Structure, Patient Characteristics, and Financial, which according to our analyses are relevant across settings and professional roles. The importance of these three particular core attributes to implementation is discussed further next.

The attribute Work Structure included features such as scheduling and shift work, teamwork, workload, standardization of care, and reminders. Several previous studies with nurses and physicians support these and similar features as important to implementation in a variety of different clinical practice settings, ranging from adult hospital and primary care settings in the UK [21], pediatric intensive care units in Canada [22], and adult and pediatric hospitals in Canada [23, 24]. Specifically, Work Structure features that have been shown to be important contextual factors for improving nurses’ use of research evidence in clinical practice include cooperation from colleagues [21, 22, 25], teamwork [23, 25], staff involvement [23, 25], relationships between staff [23, 25], and workload [22]. Similarly, Davy and colleagues [24] in a systematic review of factors influencing implementation of chronic care models by primary care practitioners (mostly general physicians) also found certain Work Structure features to be enablers of implementation: strong networks and increased communication, and regular group meetings. Additional studies, conducted within primary care with physicians, continue to find Work Structure features enable implementation. For example, Sopcak and colleagues [26] found teamwork, planning, and engaging early as a team are important contextual features enabling implementation.

The attribute Patient Characteristics included a wide range of demographic features as well as patient expectations and preferences. Previous studies in nursing and medicine, across both hospitals and primary care, support inclusion of Patient Characteristics as a core context attribute in implementation. Studies of hospital and primary care nurses in Ireland [27], hospital nurses in the USA [28], and hospital, community health, and primary care nurses in England [29] found that Patient Characteristics (e.g., demographics, attitudes, and knowledge) were related to nurses’ use of research in practice. Davy and colleagues [24] and Sopcak and colleagues [26] also found Patient Characteristics to be important to implementation by physicians in primary care, including patients’ perception of acceptability of the intervention [24], and patient resistance and/or disengagement [26]. While hospital physicians in our sample also frequently discussed Patient Characteristics as an important contextual attribute, previous studies addressing contextual factors for physicians in hospital-based settings were not located for comparison.

We also identified Financial as a core contextual attribute, evident across all behaviors, settings, and professional roles. Features within this attribute included costs (present in all datasets), financial incentives, and the funding system. Previous studies with physicians and nurses across multiple settings and countries identified Financial features and lack of financial support as important contextual attributes that restrict implementation [24, 26–28, 30–33].

### Differences by setting and role

While most of our context attributes were evident in all settings, there was variation between settings with respect to relevance and frequency of some of the finer grained features within the attributes. For example, record keeping (attribute: System Features) and financial incentives (attribute: Financial) were more frequent in primary care settings, while group make-up (attribute: Healthcare Professional Characteristics) and social interactions (attribute: Collaboration) were more frequent in hospital-based settings. This may reflect the fact that primary care settings in our sample (doctor offices and clinics) were small in comparison with the larger urban hospitals in the sample and thus have fewer staff and thus less opportunity for collaboration. Additionally, in the present study, all 14 context attributes and most features (*n* = 59, 95%) were mentioned by all groups (physicians and residents, nurses, and organ donor coordinators), indicating consistency across healthcare providers with respect to perceived attributes and features of context. Because most context attributes were common across settings and roles, we believe that a broad comprehensive context assessment instrument at the attribute level that can be used across clinical settings is possible. For a detailed context assessment, however, some slight variation may be needed in instruments in some items at the feature level, mostly with respect to primary versus hospital care.

### New attributes and features

Through this study, we identified 62 unique features of context, categorized into 14 broader attributes of context. We compared these attributes and features to the Tailored Implementation in Chronic Diseases framework (TICD) checklist [34], which is the most recent and comprehensive published consolidation of implementation frameworks that include context. The TICD includes elements that are in popular meta-typologies such as the Consolidated Framework for Implementation Research [4], as well as commonly used conceptual frameworks such as the Theoretical Domains Framework [16, 17]. Most (*N* = 13 of 14, 93%) of our attributes mapped to concepts in the TICD, supporting consensus for these 14 attributes. The one attribute from our analyses  that was not represented within TICD was Facility Characteristics, defined as the attributes of a building or group of buildings designated as a site for providing healthcare; these characteristics include, for example, type of facility (i.e., a hospital, a walk-in clinic, and a trauma center), the volume of patients cared for at that location, and the geographic location and catchment.

Of particular interest is our comparison of our more finely grained features of context to the TICD checklist. Over half (*N* = 34 of 62, 55%) of the features that emerged in our study are not contained in the TICD checklist (Additional file 2). This may be because the TICD checklist focuses more on higher-level context attributes over lower-level features. Our identification of these features represents a critical advancement in the implementation field. As a result of this work, we advance much needed conceptual clarity in context, which is critical to develop common assessment tools to measure context to determine which specific context features are more or less important in different contexts and for changing different healthcare professional behaviors. Such measurement tools can subsequently be used to (1) tailor implementation intervention designs and their delivery, (2) better interpret the effects of implementation interventions, and (3) pragmatically guide change agents and researchers in their implementation efforts.

### Strengths and limitations

The main strength of this study is that we analyzed data from 145 qualitative interviews collected using similar methods across different countries, healthcare professionals, settings, and behaviors, providing richness not available in any single dataset. A second strength is that we were able to identify attributes of context and their features across a broader range of users and settings than previous studies. Finally, the qualitative approach allowed for detailed analysis of the attributes and features of context relevant to implementation science and necessary to inform future assessments of context.

The study also has some limitations. First, despite the large number of interviews, the sample was comprised largely of physicians (78%) and healthcare professionals from hospital settings (84%). Other healthcare professionals, including allied health professionals such as rehabilitation therapists, play critical roles in clinical practice settings. Incorporating the views of these groups may provide a potentially more complete picture of the attributes of context relevant to clinical practice. Second, the relatively small proportion of international transcripts limits our study’s generalizability outside of Canada. Therefore, the attributes and features presented in this paper should be considered provisional until further validation in other countries is conducted. Finally, because this was a secondary analysis of existing qualitative data, the interviews were not designed to specifically elicit perceptions about context. However, context was explicitly recognized in the TDF that guided the interviews. Furthermore, the interview questions were broad and open-ended, allowing participants to spontaneously identify instances of context.

This study relied solely on data collected using TDF interview guides; this can be considered both a strength and a weakness. The TDF is based on 33 psychological theories that explain behavior change; context is explicitly recognized in the framework as one of its 14 theoretical domains. Interview questions were broad and open-ended, allowing participants to spontaneously identify instances of context that act as barriers and/or enablers to their use of research in clinical practice across all 14 domains. As a result, these data provided a rich array of contextual features. A limitation however of our data being based solely on the TDF is that the framework is a consolidation of physiological theories, and context is broader than psychology. Therefore, while context is one domain within the framework, it is a less robust domain in terms of conceptualization compared to the other 13 TDF domains which are inherently more psychological-based. To overcome this limitation, we assessed the full transcripts (all TDF domains) for utterances of context. Our study results can be used to refine the context domain within the TDF framework. In addition, it is important to note that the TDF is a framework and not a “theory”; therefore, relationships between its domains are not specified. As a result, it could be used to generate testable hypotheses between the context features and attributes identified in our analysis.

## Conclusion

Through this study, we identified 62 unique features of context, grouped into 14 broader attributes of context. There was considerable consistency in the 14 attributes identified irrespective of the clinical behavior, setting, or professional role, supporting broad utility of the attributes of context identified in this study. There was more variation in the finer grained features of these attributes with most variation being by setting. This is the largest study to date that has identified a large number of attributes and features of context specific to implementation. Nonetheless, further work is needed to validate this work across a broader range of settings. For example, further research should include datasets from a broad range of countries (developed as well as under-developed) and non-English speaking countries and utilize different approaches (for example, open-ended interviews, quantitative survey) to ensure that the attributes and their features identified in the present study can be generalized across other clinical behaviors, settings, and professional roles.

## Additional files

Additional file 1: Further detail on each attribute, including the identity of the features within each attribute, definitions, and an illustrative quote for each feature within the attributes, and the frequency of each feature overall and within each of the 11 datasets specifically. (XLS 129 kb)

Additional file 2: Features not mapped to TICD. Provides a listing of the 34 lower-level features of context attributes identified in this study that are not found in the TICD checklist. (DOCX 22 kb)

## Abbreviations

PARiHS: Promoting Action on Research in Health Services
TACT-A: Target, Action, Context, Time, Actor
TDF: Theoretical Domains Framework
TICD: Tailored Interventions in Chronic Diseases
