# Supplementary material for: Attributes of context relevant to healthcare professionals’ use of research evidence in clinical practice: a multi-study analysis
Source: Implement Sci. 2019 May 22;14:52. doi: 10.1186/s13012-019-0900-8 (PMC6530177; doi:10.1186/s13012-019-0900-8)
Supplement: Supplementary file 2 — Features not mapped to TICD. Provides a listing of the 34 lower-level features of context attributes identified in this study that are not found in the TICD checklist. (DOCX 22 kb) [file 13012_2019_900_MOESM2_ESM.docx]

Features not mapped top TICD

| **Attributes**  **(N=11 of 14)** | **Features (N=34 of 62)** | **Definition** |
| --- | --- | --- |
| Patient Population | Patient Demographics | Quantifiable characteristics of individuals receiving care or service including their individual or collective age, sex, weight, education, health status (number of illnesses or comorbidities, patient acuity, illness severity, etc.), medication history, and previous hospitalizations. |
| Clinician/ Provider Group | Job Autonomy | The amount of freedom a given individual, team, or professional type is given to make practice decisions and act on their clinical expertise by an organization, group, or individual in a position of authority. |
|  | Group Makeup | The overall mix of characteristics among people working at a given healthcare facility, either within a team, unit, division, or department, or as an amalgam of the entire facility workforce. This can include the combination and proportion of different professional roles, the different levels of professional experience and employee tenure at that facility, education level of employees, ages of employees, and the relative maturity or youthfulness of the practice group. |
|  | Accountability | The obligation or willingness of clinicians to accept responsibility for clinical decisions and/or professional behaviours. |
|  | Experience | Having knowledge or skill in a particular field, especially a profession or job, gained over a period of time. Often used to compare groups with different experience levels (e.g. junior residents vs. fellows). |
|  | Code of Ethics | The morals, principles, and values that an individual adheres to, which may define their interactions with others, and may be influenced by both personal and published professional codes of ethics. |
| Work Structure | Continuity of Care | Consists of both patient and provider experiences. For a patient it is the experience of a 'continuous caring relationship' (sustained contact between a patient and provider over time) with an identified healthcare professional or a network of healthcare providers. For providers, it is the delivery of a 'seamless service' through integration, coordination, and the sharing of information between different providers. |
|  | Standardization of Care | Efforts to coordinate and systematize the work practices, information, and care procedures required for the delivery of healthcare. |
|  | Workload | The amount of work to be performed as it is distributed across a clinical team |
|  | Scheduling | Designated work times, the arrangement of work times among a clinical team (including shift work), and other on-call arrangements. |
|  | Delegation of Tasks | The assignment of responsibility or authority to another person (normally from a manger to a subordinate) to carry out specific activities. |
|  | Reminders | Memory aids and other environmental cues intended to facilitate the change or act as a cue for a particular clinical behaviour or set of clinical behaviours. |
| Facility Characteristics | Type of Facility | The practice setting where a clinician or team of clinicians operates. This can include private clinics, hospitals, nursing care homes, public health practices, client homes (e.g. home care) and specialty practices (e.g. The Heart Institute, The Ottawa Integrative Cancer Centre, etc.). |
|  | Geography | The location and situation of a healthcare facility. This can refer to the country (or region, province, state, etc.), where the facility is located (including multiple sites), and the characteristics of the locale(s) (e.g. rural, urban, etc.). |
|  | Programs | A planned, sequential process of teaching and learning provided to patients and clients for the purpose of addressing a particular healthcare issue (e.g. smoking, drug-use, diet, etc.). |
|  | Volume | Quantity of patients that are cared for in a particular organization within a certain timeframe. |
|  | Atmosphere | The encompassing tone or mood of a place or situation in a healthcare facility as influenced by environmental cues (e.g. lighting, noise level), the design of space, and social climate (e.g. tension, calm demeanors), affecting the experience of the patient or healthcare worker. |
|  | Size | The relative proportion or magnitude of a given unit or facility, often measured by number of staff, number of patients served, number of beds, and/or number of units. The size of a given facility is often related to its geography and its rural/urban status, but it should be kept in mind that a rural hospital can be a 'large' rural hospital, and an urban hospital could be considered proportionally small. Facility size is, therefore, here considered to be a relative concept. |
| Leadership | Mentorship | A relationship established between a leader or superior and a subordinate or trainee, characterized by a close, and typically enduring, pedagogical exchange, where the subordinate or trainee learns by observing and regularly communicating with the leader or superior. |
| Financial | Funding System | A configuration of services that varies from country to country, but in all cases consists of a financing mechanism; a paid workforce; information on which to base decisions and policies; facilities; and logistics to deliver quality medicines and technologies. |
|  |  |  |
| Resource Access | Documentation | Digital or printed documents (including handwritten notes or checkboxes filled out on pre-printed forms) used by multidisciplinary team members to record medical treatments, medical tests, patient interactions, observations, and plans of treatment. These documents can be referenced by interdisciplinary team members to provide guidance in clinical practice, as they show a record of what has been done. |
|  | Resource Quality | A quality resource is one which has any number of the following characteristics: useful, cost effective, readable, consistent, interpretable, empirically validated, reliable, specific, applicable, and/or up to date. |
|  | Guidelines | Systematically developed statements (and diagnostic decision aids) designed to assist practitioner and patient decisions about appropriate healthcare for specific clinical circumstances. Guidelines are a discretionary set of practice recommendations, rather than a binding set of mandates. |
|  | Proximity | The relative distance (i.e. close or far) of resources of whatever kind (e.g. objects, people, equipment), potentially prompting the remembrance of a particular practice behaviour. |
|  | Technology | Systems, software, and devices which are the result of scientific knowledge being applied for the purpose of providing health services. This includes electronic documentation systems, innovative medical devices (e.g. laparoscopic operating systems), tablets/PDAs and other similarly sophisticated medical devices. |
|  | Space as a Resource | The presence/absence, design, maintenance, and allocation of areas that are properly equipped, clean, and of sufficient size and number for the provision of health care in a facility. |
|  | Online Resources | Readily accessible websites, data-bases, and/or other information sources available to a clinician or patient by means of the internet or other electronic network. |
| System Features | Resource Waste | The excessive use, consumption, or expense of resources. |
|  | Logistics & Coordination | The work that must be done to plan and organize a complicated activity or event, often involving many people. This consists of "the deliberate organization of...care activities between two or more participants...to facilitate the appropriate personnel and other resources needed to carry out" healthcare services. |
| Collaboration | Social Interactions | Informal influences arising from a clinician's relationship with others in their work environment, including colleagues, patients, and patients' families. To be considered for inclusion, such interactions must take place outside of communication intended to facilitate the work process (e.g. rounds, audit feedback, team conversations, etc.), but can include discussions that take place during patient-clinician consultations. |
| Evaluation | Audit | The official inspection of a division, department, or clinician group, typically by an independent body. |
|  | Organizational Evaluation | Assessments carried out by an organization in order to obtain data on employee/system performance, identify barriers and facilitators to achieving organizational goals, and provide guidance to staff members. |
|  | Patient Evaluation | Evaluative comments or feedback to health care professionals made by patients and their families. This can include: formal surveys, questionnaires, interviews, and informal conversations. |
| Societal Influences | Societal Influences | The general level of social knowledge and attitude as it regards to a particular clinical behaviour or procedure. For example, widespread attitudes about organ donation, or a public reaction to a hospital audit as it has been portrayed in the media. |
